# Conical Implants in Tuberous Breast Correction: Clinical and Patient-Reported Outcomes

**DOI:** 10.3390/medicina62050930

**Published:** 2026-05-10

**Authors:** Jorge González-Prieto, Antonio González-Nicolás, Barbara Helena Barcaro Machado

**Affiliations:** 1 Department of Plastic Surgery, Hospital Universitario de Getafe, 28905 Madrid, Spain; 2 Private Practice, 28006 Madrid, Spain; gnicolas@drgnicolas.com; 3 Private Practice, Rio de Janeiro 22430-041, Brazil; barbara@barbaramachado.com.br

**Keywords:** tuberous breast deformity, conical implants, breast augmentation, polyurethane implants, BREAST-Q, aesthetic breast surgery

## Abstract

*Background and Objectives*: Tuberous breast is a complex congenital deformity that requires correction of the underlying stenotic anatomy. Clinical evidence on the use of conical polyurethane implants in this setting is limited. To evaluate clinical outcomes and patient-reported satisfaction following correction of tuberous breast deformity using a standardized, implant-assisted reconstructive protocol with exclusive use of conical polyurethane implants. *Materials and Methods*: An ambispective study included 50 patients with tuberous breast deformity treated between 2020 and 2025 by two surgeons using a standardized implant-assisted reconstructive protocol. All patients underwent systematic glandular ring release and inframammary fold repositioning, followed by placement of conical polyurethane implants. Outcomes included complications, reoperations, and BREAST-Q Augmentation V2.0 scores. The mean follow-up was 17 months (range, 9–24 months). *Results*: The mean patient age was 29.8 years. According to the Grolleau classification, 62% of patients were type I, 30% type II, and 8% type III. The mean implant volume was 258.2 cc. Overall complication rate was 10%, including one case (2%) of capsular contracture secondary to infection, with a reoperation rate of 8%. Postoperative BREAST-Q scores showed high levels of patient satisfaction, with mean “satisfaction with breasts” scores of 90.1 ± 11.9 and 92.0 ± 9.7 at the first and second postoperative assessments, respectively. *Conclusions*: Within a standardized reconstructive protocol, conical polyurethane implants were associated with high postoperative patient satisfaction and acceptable complication rates during early-to-mid-term follow-up in the correction of tuberous breast deformity. These findings suggest that the use of conical polyurethane implants within a standardized reconstructive approach is feasible in selected cases. Further comparative studies with longer follow-up are warranted.

## 1. Introduction

### 1.1. Anatomical Basis and Classification

Tuberous breast, also referred to as tubular breast or nipple–areolar complex (NAC) deformity, is a congenital anomaly of breast development first described by Rees and Aston in 1976 [1]. It is characterized by a constricted breast base, glandular hypoplasia, an elevated inframammary fold, areolar hypertrophy, and glandular herniation into the areola, frequently associated with breast asymmetry [2,3,4,5,6,7]. Although its exact prevalence remains uncertain, estimates suggest it may affect 6–73% of patients undergoing breast surgery, depending on the diagnostic criteria and classification system applied [2,8].

From an anatomical standpoint, the deformity has been attributed to several factors, including thickening of the superficial fascial system and the presence of a constrictive fibrous ring in the lower breast pole, which impedes normal peripheral glandular expansion during puberty [2,7,8,9,10,11,12]. Additionally, adhesions between the dermis and pectoral muscle in the lower quadrants, as well as histological increases in collagen and elastic fibers within the superficial fascia, have been reported. These alterations contribute to parenchymal hypoplasia, breast ptosis, and the distinctive cylindrical breast shape [3,8].

To classify the severity of the deformity, Grolleau [13] proposed a widely adopted three-tier system (types I, II, and III), based on the extent of hypoplasia in the lower quadrants. This system simplified the earlier classification developed by von Heimburg [14]. Complementing these morphological classifications, the Northwood Index provides a quantitative tool for assessing areolar protrusion in relation to its diameter, facilitating the identification of mild or underdiagnosed cases [15].

Beyond traditional classification systems, Klinger et al. proposed the concept of stenotic breast malformation as a continuous anatomical spectrum, ranging from mild constriction deformities to fully developed tuberous breasts, rather than discrete entities [16]. This framework broadens the understanding of the underlying pathoanatomy by emphasizing that varying degrees of base constriction, glandular herniation, and inframammary fold abnormalities may coexist across a continuum of severity. Importantly, this perspective underscores the need for individualized surgical strategies tailored to the specific anatomical features of each patient, rather than a one-size-fits-all approach, particularly when planning reconstructive strategies aimed at correcting lower pole deficiency and breast base constriction.

### 1.2. Therapeutic Options

The surgical correction of tuberous breasts remains a complex challenge due to the anatomical features characteristic of this deformity, including a constricted breast base, hypoplastic lower pole, elevated inframammary fold, and herniation of the NAC. Traditional approaches typically involve a combination of procedures such as fibrous ring release, glandular remodeling, fat grafting, and the placement of round or anatomical implants [2,8].

Among the classic glandular remodeling techniques, the flaps described by Ribeiro [17] and Puckett [18,19] are frequently employed to redistribute available glandular tissue into the lower pole. However, their effectiveness is limited in cases with pronounced glandular hypoplasia, particularly in Grolleau types II and III. In such instances, adjunctive procedures such as rigotomies, autologous fat grafting, or the use of tissue expanders are often necessary to achieve adequate lower pole volume or expansion [19,20,21,22].

Correction of the elevated inframammary fold typically involves surgical repositioning through controlled dissection. However, in patients with significant “tissue memory,” preliminary tissue expansion may be required, increasing the overall complexity of treatment [5,8,13]. Areolar herniation is commonly addressed using techniques such as circumareolar de-epithelialization and periareolar “round block” closure (Benelli [23] or Hammond [24]), which helps reduce the areolar diameter and reposition the NAC. However, they do not directly correct the deficient lower pole.

In recent years, lipofilling has gained traction as a complementary technique, offering improvements in contour and correction of deformities such as glandular hypoplasia, double-bubble deformity, or noticeable transitions between implant and breast tissue [11,20,21,25,26]. However, its use is limited by the need for adequate donor fat, the potential for variable resorption, and the requirement for multiple sessions.

Despite technical advances, conventional round and anatomical implants may fall short in achieving sufficient lower pole expansion, particularly in more severe deformities. This situation can lead to residual deformities, an increased risk of reoperations, and greater surgical complexity [6,13,27].

In response to these limitations, conical implants have emerged as a potential option. Their geometry may facilitate progressive lower pole expansion, enhanced projection, and a smoother transition between implant and native tissue, making them a potentially valuable adjunct within an implant-assisted reconstructive strategy [28]. However, to date, no clinical studies have systematically evaluated the exclusive use of conical implants for the correction of tuberous breast deformities.

To our knowledge, this is the first clinical study to report the exclusive use of conically shaped implants across the spectrum of tuberous breast deformity grades. The present study is intended to provide initial clinical evidence within this specific implant-assisted approach rather than to propose a fundamentally new surgical technique. The aim of this study was to evaluate clinical outcomes, complication rates, and patient-reported outcomes in patients with tuberous breast deformity treated using conical polyurethane implants using a standardized surgical protocol.

## 2. Materials and Methods

This observational, descriptive, and ambispective study included 50 patients clinically diagnosed with tuberous breast deformity who underwent corrective surgery between November 2020 and January 2025. Written informed consent was obtained from all participants. The study protocol was reviewed and approved by the Ethics Committee of Hospital Universitario de Getafe (CEIm25/82) under the title “Conical Implants in Tuberous Breasts: An Ambispective Study”.

All procedures were performed by two plastic surgeons with extensive experience in aesthetic and reconstructive breast surgery following the same standardized protocol. Both surgeons applied the same core reconstructive principles in all cases (systematic glandular ring release and standardized nipple–areola complex management), while adjunctive steps followed predefined anatomical criteria within the same protocol.

Surgical and perioperative data were collected retrospectively from medical records, while postoperative outcomes were assessed prospectively using a standardized follow-up protocol applied to all patients. Clinical variables collected included patient age, tuberous breast classification, smoking status, surgical approach, implant volume and projection, nipple–areola complex management, and the occurrence of complications or reoperations.

### 2.1. Inclusion and Exclusion Criteria

The study included adult female patients with a clinical diagnosis of tuberous breast according to the Grolleau classification (types I, II, or III) who had no prior history of breast surgery and agreed to complete postoperative follow-up, including the BREAST-Q Augmentation V2.0 questionnaire. Type III deformities were included for completeness and descriptive purposes but were not analyzed as an independent subgroup due to the limited sample size. Patients presenting mixed breast deformities, those with previous breast interventions, or those who failed to complete follow-up evaluations were excluded.

### 2.2. Surgical Technique

All patients received conical polyurethane implants (Silimed Advance^®^, SILIMED Indústria de Implantes Ltda., Rio de Janeiro, Brazil) without the use of fat grafting or tissue expanders.

Implant selection was guided by anatomical parameters, including native breast base width, degree of lower pole constriction, soft-tissue envelope characteristics, and the desired postoperative breast base and projection, rather than a fixed algorithm. A standardized implant-assisted reconstructive protocol with predefined anatomical adaptations was applied across all cases, based on consistent core surgical principles with predefined modifications according to deformity severity. The periareolar approach was used as the primary surgical access in all patients, including complete or inferior hemiperiareolar incisions depending on anatomical requirements. When additional skin envelope reshaping was required due to ptosis or significant lower pole skin excess, the periareolar incision was combined with mastopexy. No inframammary approach was used in any case.

Glandular ring release was systematically performed in all patients and consisted of radial glandular scoring and deep parenchymal detachment to correct the underlying constricted breast base and facilitate lower pole expansion. The release was extended through the full thickness of the constricted glandular tissue until adequate mobilization of the lower pole was achieved, while preserving overlying skin viability. Lower pole expansion was achieved through multiple radial glandular incisions, typically ranging from three to five releases depending on tissue stiffness and deformity severity. In addition, the inframammary fold was released and repositioned in all cases to eliminate the constrictive memory of the breast base and allow appropriate lower pole development [22].

Implant pockets were created using a predominantly dual-plane II technique, with partial release of the pectoralis major muscle to optimize lower pole expansion while maintaining upper pole coverage. In selected cases with adequate soft-tissue coverage and minimal upper pole constriction, a subglandular pocket was used according to predefined anatomical criteria. Posterior myotomy was performed as needed to facilitate lower pole expansion, with the use of hydrogen peroxide–soaked sponges to assist muscle release and hemostasis.

The Puckett parenchymal flap technique [18,19] was selectively applied in Grolleau type I and II deformities when sufficient glandular tissue allowed effective redistribution toward the lower pole. In type II and III deformities with limited parenchymal availability, correction relied on glandular release combined with implant-assisted expansion. Management of the nipple–areola complex was standardized in all cases using a circumareolar Benelli technique [23], allowing controlled areolar reduction and repositioning.

Prior to implant insertion, both the implant and the pocket were irrigated with an antibiotic solution consisting of saline with gentamicin and cefazolin. Implant orientation was confirmed using the manufacturer’s orientation marker to align the implant apex with the nipple–areola complex. No staged reconstruction, acellular dermal matrices, or adjunctive fat grafting were used in any case.

Postoperative follow-up ranged from 9 to 24 months, with shorter follow-up corresponding to the most recently treated patients. Follow-up included regular clinical examinations and standardized photographic documentation. Standardized clinical photographs were obtained at predefined postoperative time points to illustrate the temporal evolution of the reconstruction, including early postoperative stages. However, clinical outcomes and patient-reported outcome measures were evaluated exclusively after a minimum follow-up of 9 months, in accordance with the study protocol.

Preoperative markings followed a standardized anatomical protocol and are demonstrated in the accompanying surgical video (Video S1), which illustrates the key steps of surgical planning and execution.

### 2.3. Outcome Assessment

Outcomes were evaluated using both objective and subjective measures:

- Objective clinical parameters: incidence of complications (e.g., implant extrusion, dehiscence, capsular contracture, implant replacement) and need for reoperation. Reoperations were defined as any secondary surgical procedure requiring a return to the operating room, including implant exchange, capsulotomy/capsulectomy, NAC revision, or scar revision. Reoperations included both procedures performed to address complications and elective aesthetic revisions, such as secondary fat grafting.
- Patient-reported outcomes: via the BREAST-Q Augmentation V2.0 postoperative questionnaire [29,30,31].

The BREAST-Q was self-administered online or completed via structured telephone interview when internet access was limited. The following domains were analyzed: satisfaction with breasts (two questionnaires), physical well-being of the chest (two questionnaires), and satisfaction with implants. Raw scores were transformed to a 0–100 scale. Higher scores indicated more favorable outcomes for positively framed domains. For negatively framed items assessing symptoms (e.g., pain, tightness, asymmetry, wrinkling), lower scores reflected better results.

### 2.4. Statistical Analysis

Statistical analysis was primarily descriptive. Continuous variables are presented as means and standard deviations, and categorical variables as frequencies and percentages. Given the observational and non-comparative nature of the study, no formal inferential statistical analysis was performed.

## 3. Results

### 3.1. Patient Demographics and Surgical Characteristics

A total of 50 patients with a clinical diagnosis of tuberous breast deformity were included in the study. All patients underwent surgical correction using conical polyurethane implants within a standardized reconstructive protocol with predefined anatomical adaptations. Postoperative follow-up ranged from 9 to 24 months, with a mean follow-up of approximately 17 months. The mean patient age was 29.8 years (range, 18–58 years). Only one patient (2%) reported active tobacco use, while the remaining patients were non-smokers (Table 1).

**Figure 1:**
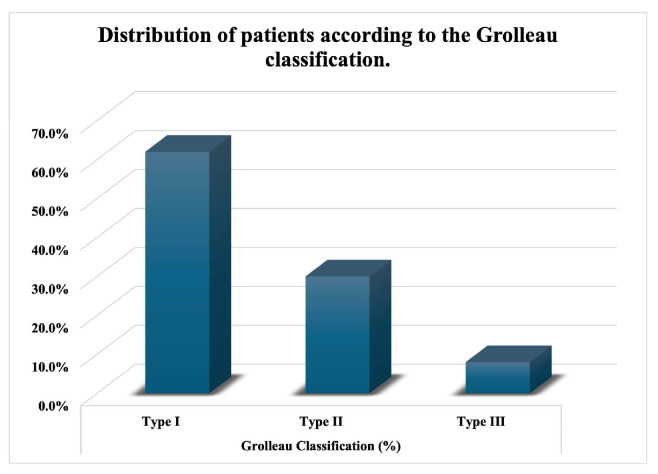
Distribution of patients according to the Grolleau classification.

According to the Grolleau classification, 31 patients (62%) were classified as type I, 15 patients (30%) as type II, and 4 patients (8%) as type III (Figure 1).

The mean implant volume was 258.2 cc, ranging from 125 to 410 cc (Table 1).

**Figure 2:**
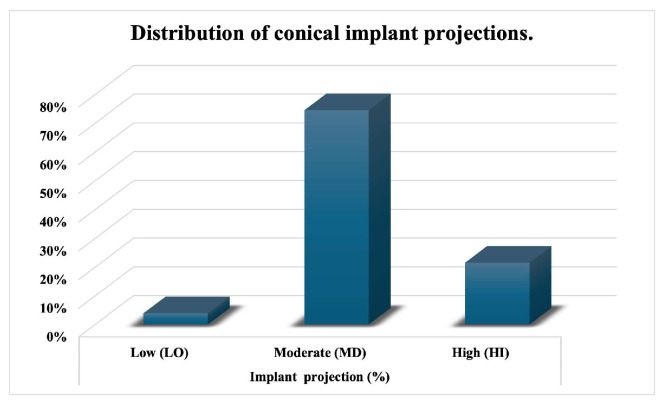
Distribution of conical implant projections.

The most used implant projection was moderate (MD), applied in 74% of cases, followed by high projection (HI) in 22%, and low projection (LO) in 4% (Figure 2).

The periareolar approach was used as the primary surgical access in all patients, including complete or inferior hemiperiareolar incisions depending on anatomical requirements. A periareolar-only approach was sufficient in 34 patients (68%), whereas a combined periareolar approach with mastopexy was required in 16 patients (32%) due to ptosis or significant lower pole skin excess. No inframammary surgical access was used in this series.

Glandular ring release was systematically performed in all patients as part of the standardized reconstructive protocol. The Puckett parenchymal flap technique [18,19] was selectively applied in Grolleau type I deformities when sufficient glandular tissue allowed effective redistribution toward the lower pole, whereas in type II and III deformities, correction relied on glandular release combined with implant-assisted expansion.

Nipple–areola complex management was performed according to the standardized protocol described in the Methods section. NAC position and symmetry were assessed clinically and through standardized photographic documentation. Objective anthropometric measurements were not routinely collected, which represents a limitation of this study. No secondary procedures for scar revision or nipple–areola complex revision were required during the follow-up period.

**Figure 3:**
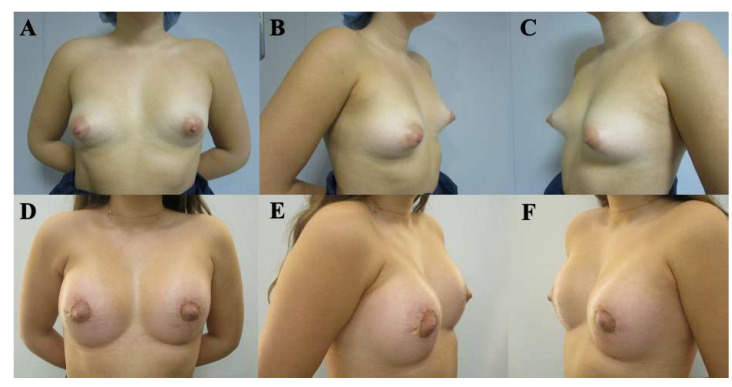
Preoperative and postoperative clinical photographs of a 26-year-old female patient presenting with bilateral tuberous breast deformity (Grolleau type II). Surgical correction was performed using a standardized implant-assisted reconstructive protocol, including circumferential glandular ring release, inframammary fold release and repositioning, and parenchymal reshaping using the Puckett technique, through a periareolar approach. Bilateral conical polyurethane implants (295 cc, moderate projection [MD]) were placed in a dual-plane II position. Preoperative views (**A**–**C**) and early postoperative views at 2 months (**D**–**F**) are shown, including frontal (**A**,**D**), left oblique (**B**,**E**), and right oblique (**C**,**F**) perspectives. Early postoperative images (obtained before 6 months of follow-up) are included exclusively to illustrate the temporal evolution of reconstruction and were not used for outcome assessment. These images represent selected illustrative cases and are not intended to reflect the full range of outcomes observed in the study cohort.

**Figure 4:**
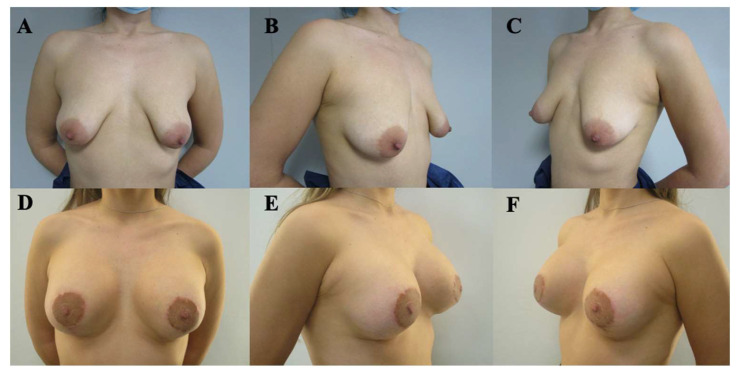
Preoperative and postoperative clinical photographs of a 25-year-old female patient presenting with bilateral tuberous breast deformity (Grolleau type III). Surgical correction was performed using a standardized implant-assisted reconstructive protocol, including circumferential glandular ring release and inframammary fold repositioning, combined with mastopexy. Bilateral conical polyurethane implants (375 cc, moderate projection [MD]) were placed in a dual-plane II position. Preoperative views (**A**–**C**) and postoperative views at 12 months (**D**–**F**) are shown, including frontal (**A**,**D**), left oblique (**B**,**E**), and right oblique (**C**,**F**) perspectives. These images represent selected illustrative cases and are not intended to reflect the full range of outcomes observed in the study cohort.

**Figure 5:**
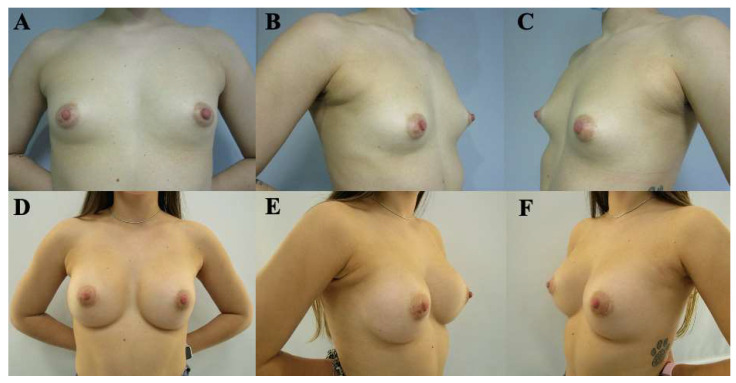
Preoperative and postoperative clinical photographs of a 31-year-old female patient presenting with bilateral tuberous breast deformity (Grolleau type I). Surgical correction was performed using a standardized implant-assisted reconstructive protocol, including circumferential glandular ring release, inframammary fold repositioning, and parenchymal reshaping using the Puckett technique, through a periareolar approach. Bilateral conical polyurethane implants (325 cc, moderate projection [MD]) were placed in a dual-plane II position. Preoperative views (**A**–**C**) and postoperative views at 18 months (**D**–**F**) are shown, including frontal (**A**,**D**), left oblique (**B**,**E**), and right oblique (**C**,**F**) perspectives. These images represent selected illustrative cases and are not intended to reflect the full range of outcomes observed in the study cohort.

**Figure 6:**
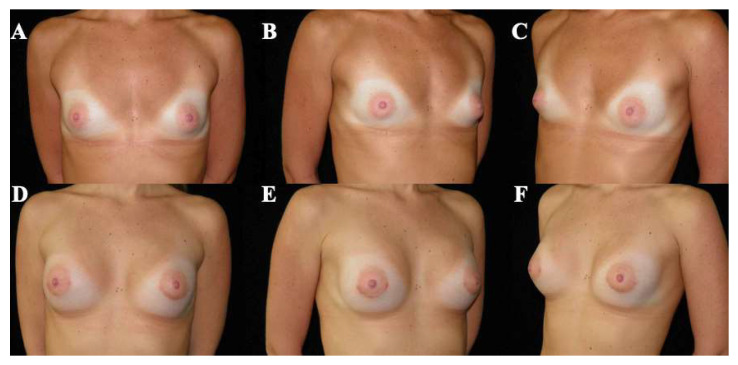
Preoperative and postoperative clinical photographs of a 45-year-old female patient presenting with bilateral tuberous breast deformity (Grolleau type II). Surgical correction was performed using a standardized implant-assisted reconstructive protocol, including circumferential glandular ring release, inframammary fold repositioning, and parenchymal reshaping using the Puckett technique, through a periareolar approach. Bilateral conical polyurethane implants (210 cc, moderate projection [MD]) were placed in a dual-plane II position. Preoperative views (**A**–**C**) and postoperative views at 24 months (**D**–**F**) are shown, including frontal (**A**,**D**), left oblique (**B**,**E**), and right oblique (**C**,**F**) perspectives. These images represent selected illustrative cases and are not intended to reflect the full range of outcomes observed in the study cohort.

**Figure 7:**
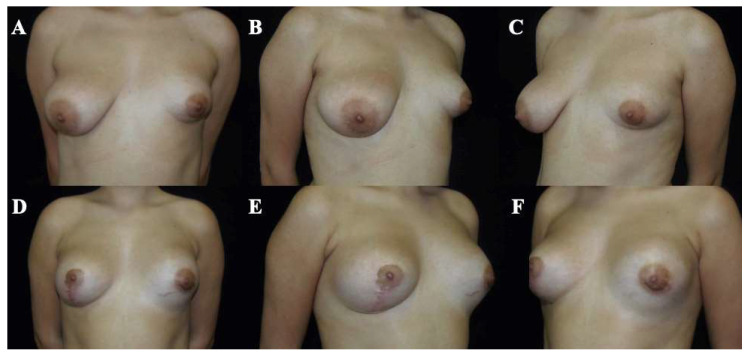
Preoperative and postoperative clinical photographs of a 38-year-old female patient presenting with bilateral tuberous breast deformity (Grolleau type III). Surgical correction was performed using an implant-assisted reconstructive approach, including circumferential release of the constricted glandular ring, inframammary fold repositioning, and parenchymal reshaping. A mastopexy was performed on the right breast, while a periareolar approach was used on the left breast. Bilateral conical polyurethane implants (325 cc, moderate projection [MD]) were placed in a Dual Plane II position. Preoperative views (**A**–**C**) and postoperative views at 24 months (**D**–**F**) are shown, including frontal (**A**,**D**), left oblique (**B**,**E**), and right oblique (**C**,**F**) perspectives. These images represent selected illustrative cases and are not intended to reflect the full range of outcomes observed in the study cohort.

Postoperative evolution was documented using standardized clinical photography obtained at predefined, progressive postoperative time points, allowing visualization of the temporal course of healing and breast adaptation (Figure 3, Figure 4, Figure 5, Figure 6 and Figure 7). These cases are presented for illustrative purposes only and do not necessarily represent the full cohort. Early postoperative images are presented exclusively for illustrative purposes and do not represent the final outcomes analyzed in this study. Final outcome evaluation was based on longer-term postoperative photographs of the same patients, obtained after a minimum follow-up of 9 months, although not all of these images are shown for illustrative purposes.

At 2 months postoperatively (Figure 3), early tissue adaptation was observed, characterized by progressive implant settling and initial lower pole expansion.

By 12 months (Figure 4), the breasts exhibited improved contour definition with continued soft tissue remodeling and stabilization of the inframammary fold.

At 18 months (Figure 5), breast contour appeared maintained, with persistent inframammary fold definition and lower pole development.

Figure 6 and Figure 7 illustrate the late postoperative results at 24 months, demonstrating maintained breast shape and projection during the available follow-up period.

### 3.2. Patient-Reported Outcomes (Breast-Q Questionnaire)

Subjective outcomes were assessed using the postoperative BREAST-Q Augmentation V2.0 questionnaire, showing high levels of postoperative patient satisfaction and favorable functional outcomes (Table 2).

In the “satisfaction with breasts” domain, mean scores were 90.1 and 92.0 at the first and second postoperative assessments, respectively (scale: 0–100), reflecting high levels of postoperative satisfaction.

In the “physical chest well-being” domain, mean scores were 7.6 at the first assessment and 6.0 at the second. As this domain reflects symptom burden, lower scores indicate better physical well-being, indicating low symptom burden and good postoperative tolerance.

In the “satisfaction with implants” domain, the mean score was 7.8 on the original BREAST-Q scale (range: 2–8), corresponding to approximately 96 on the 0–100 Q-score scale, indicating a high level of patient satisfaction with the implant choice and overall surgical outcome. The original 2–8 scale is provided for reference, while values are presented on the standardized 0–100 scale for consistency.

### 3.3. Complications

The overall postoperative complication rate was 10% (*n* = 5) (Table 3).

One early implant-related complication (2%) occurred during hospitalization and consisted of an infection associated with a fluid collection, which required implant replacement. Additionally, three cases (6%) of minor surgical wound dehiscence were observed during the early postoperative period; these were successfully managed with conservative local wound care and healed without further intervention. During postoperative follow-up, one additional implant-related complication was identified (2%), consisting of capsular contracture. No cases of implant rupture were observed throughout the follow-up period. The overall reoperation rate was 8% (n = 4), including one reoperation for capsular contracture, two elective secondary fat grafting procedures to improve aesthetic contour, and one implant replacement related to infection.

## 4. Discussion

Tuberous breast remains one of the most challenging congenital deformities in aesthetic breast surgery due to its complex morphology and the technical constraints imposed by a constricted breast base, hypoplasia of the lower pole, an elevated inframammary fold, and herniation of the nipple–areola complex [2,5,9,20,25]. These anatomical features frequently require multifaceted reconstructive strategies to achieve satisfactory aesthetic and functional outcomes. In this ambispective series, correction performed within a standardized implant-assisted reconstructive protocol using conical polyurethane implants was associated with high postoperative patient satisfaction, acceptable complication rates, and a low reoperation rate within the observed follow-up period. Given the limited number of type III cases, the present findings are primarily applicable to Grolleau type I–II deformities. Therefore, the applicability of these findings to severe deformities remains limited.

Traditional corrective approaches—typically combining fibrous ring release, glandular remodeling, and lipofilling—may be insufficient in selected cases and can require staged procedures [2,5,9,20,25] or additional adjunctive maneuvers, increasing surgical complexity and the likelihood of secondary interventions [6,13,32]. Implant selection therefore plays a relevant role within the reconstructive algorithm, particularly when addressing a narrow and stenotic breast footprint. The geometric interaction between implant shape and a constricted mammary base is particularly relevant in tuberous deformity.

Round implants achieve projection largely through proportional increases in base diameter, which may generate greater peripheral tension within a restricted envelope. Anatomical implants distribute projection asymmetrically; however, their predefined lower pole contour still requires adequate footprint accommodation and may present rotational concerns in certain contexts [11,33].

Conical implants are characterized by a high projection-to-base ratio, concentrating volume centrally while maintaining a relatively contained base width. In the context of a surgically released but still restrictive soft-tissue envelope, this configuration may facilitate adaptation within a narrow breast footprint without excessive lateral expansion.

From a biomechanical perspective, limiting peripheral stretch may reduce lateral tension while directing volumetric expansion toward the inferior pole regions created through systematic glandular scoring and inframammary fold repositioning. This interaction may contribute to progressive lower pole filling and soft-tissue redraping during postoperative settling. The polyurethane coating may further contribute to implant stability during early tissue adaptation, which could be relevant when maintaining a newly defined inframammary fold.

However, these biomechanical considerations remain theoretical and were not directly validated within the present study. Therefore, the proposed role of conical implants in facilitating lower pole expansion should be interpreted as hypothesis-generating rather than definitively demonstrated by the present data.

In this series, capsular contracture occurred in a single patient (2%), secondary to postoperative infection rather than as a primary implant-related complication [5,12,34].

Importantly, implant geometry does not replace meticulous anatomical correction and should not be interpreted as inherently superior to other implant types. Rather, within an anatomy-driven reconstructive protocol grounded in systematic glandular release and inframammary fold repositioning, conical implants may represent a facilitating option in selected patients with mild-to-moderate deformities and a narrow breast base.

In our experience, this standardized implant-assisted approach was reproducible in patients with mild-to-moderate tuberous breast deformities. Importantly, outcomes are multifactorial and primarily depend on adequate anatomical correction rather than implant design alone. The findings reinforce that successful correction depends primarily on adequate anatomical release, with implant geometry serving as an adjunctive component within a comprehensive reconstructive strategy rather than as an isolated determinant of outcome.

### 4.1. Patient-Reported Outcomes

Patient-reported outcomes assessed using the BREAST-Q Augmentation V2.0 questionnaire showed high postoperative levels of satisfaction with breast appearance and implant-related outcomes [29,30,31]. Favorable physical chest well-being scores suggest good postoperative tolerance and functional recovery. These findings are clinically relevant in a condition known to negatively affect body image and self-esteem, reinforcing the importance of incorporating validated patient-reported outcome measures alongside objective clinical parameters.

However, given the absence of preoperative BREAST-Q assessment, these findings should be interpreted as descriptive postoperative outcomes and do not allow evaluation of changes over time.

### 4.2. Safety Profile and Reoperations

The overall complication rate (10%) and reoperation rate (8%) observed in this series appear favorable when compared with reports involving combined or staged reconstructive techniques, although direct comparisons are limited by differences in study design, patient selection, and follow-up duration. Capsular contracture and postoperative infection represented the implant-related events identified during follow-up, with one case each. No cases of implant rupture were observed [35,36].

Despite frequent concerns regarding periareolar scarring in tuberous breast correction, no secondary procedures for scar revision or nipple–areola complex revision were required during the follow-up period in this cohort.

### 4.3. Standardization and Reproducibility

A major strength of this study lies in the consistent application of a standardized reconstructive protocol by two experienced surgeons practicing in different geographic and healthcare settings, which may support the external validity of the present findings. Across all cases, the fundamental steps of tuberous breast correction were systematically performed, including glandular ring release, release and repositioning of the inframammary fold, correction of the constricted breast base, and standardized circumareolar management of the nipple–areola complex using the Benelli technique.

The periareolar approach was uniformly adopted as the primary surgical access, with mastopexy reserved exclusively for predefined anatomical indications. Adjunctive maneuvers, such as the selective use of the Puckett parenchymal flap technique in Grolleau type I deformities with sufficient glandular tissue, were applied according to explicit anatomical criteria rather than surgeon preference.

Importantly, this standardized protocol does not rely on highly specialized equipment or complex staged procedures, supporting its reproducibility in routine clinical practice. While implant geometry alone cannot be isolated as the sole determinant of outcome, the present series evaluates clinical and patient-reported results within a reproducible, implant-assisted reconstructive framework driven by anatomical correction. In this context, conical implants should be regarded as a facilitating adjunct within a comprehensive, anatomically based surgical strategy, rather than as a standalone solution replacing established reconstructive principles.

Within this framework, the selective presentation of clinical images was intentional and aimed at illustrating key aspects of surgical correction, while outcome assessment relied on longer-term follow-up images reviewed for all patients.

### 4.4. Clinical Implications and Future Directions

The present findings provide preliminary clinical evidence suggesting the use of conical implants within an implant-assisted standardized protocol for tuberous breast correction, particularly in mild-to-moderate deformities. Future studies incorporating comparative designs and longer follow-up will be required to further define durability of results and long-term capsular behavior. For surgeons managing mild-to-moderate tuberous breast deformities, this implant-assisted standardized approach represents a pragmatic option that balances anatomical correction, patient satisfaction, and surgical simplicity.

### 4.5. Limitations

Several limitations of this study should be acknowledged. First, the ambispective design and the absence of a control group—such as cohorts treated with round or anatomical implants, or hybrid reconstructive techniques—limit the ability to draw comparative conclusions regarding implant superiority or a reduced need for adjunctive surgical maneuvers [8,37,38]. Accordingly, the present study was not designed to establish comparative effectiveness, but rather to report clinical and patient-reported outcomes achieved within a defined implant-assisted reconstructive protocol. Given the descriptive, non-comparative nature of this case series and the absence of a predefined primary comparative endpoint, a formal a priori power analysis was not performed, and the study was not powered to detect small differences between deformity grades or surgical variations. Accordingly, this study should be interpreted as a descriptive case series, and no causal inference or comparative conclusions regarding implant superiority can be established. Future comparative studies evaluating different implant geometries may help clarify the independent contribution of implant shape to clinical outcomes.

Second, although the mean postoperative follow-up was approximately 17 months, inclusion of the most recently treated patients resulted in a minimum follow-up of 9 months. Long-term outcomes, including capsular contracture and contour-related deformities such as double bubble, cannot be definitively assessed within the current follow-up period. This consideration is particularly relevant for polyurethane-coated implants, for which extended observation periods are required to fully assess late capsular dynamics [35,36]. Future studies incorporating longer follow-up durations and comparative designs will be necessary to clarify these aspects.

Third, the relatively small number of patients with Grolleau type III deformities precluded meaningful subgroup analysis in more severe phenotypes. As a result, the present findings are primarily applicable to mild-to-moderate tuberous breast deformities (types I–II), and extrapolation of these results to more severe cases should be performed with caution. Larger series or multicenter studies will be required to better define outcomes in advanced deformities.

Fourth, objective anthropometric measurements were not routinely collected. In particular, no objective measurement of lower pole expansion was performed, limiting quantitative evaluation of this specific outcome. Instead, the study prioritized patient-reported outcome measures (PROMs), assessed using the BREAST-Q, reflecting the growing recognition of patients’ subjective perception of symmetry, satisfaction, and overall well-being as relevant indicators of surgical success, particularly in conditions with a significant psychosocial impact.

A further consideration relates to implant selection. Although implant choice was guided by consistent anatomical parameters, including breast base width, degree of lower pole constriction, and soft-tissue characteristics, the absence of a strictly defined selection algorithm may limit reproducibility across surgeons and clinical settings. This approach reflects the need for individualized surgical planning in tuberous breast deformity, where implant selection is tailored to patient-specific anatomical features within a standardized reconstructive framework.

The absence of preoperative BREAST-Q assessment represents an additional limitation, as it precludes evaluation of changes over time and restricts interpretation of the magnitude of patient-reported improvements. Furthermore, in some cases the BREAST-Q was administered via structured telephone interviews rather than self-completed online questionnaires, which may introduce interviewer bias and potentially influence response patterns.

An exploratory independent external aesthetic assessment was also performed on a selected subset of representative photographic cases by five independent plastic surgeons using a Likert scale. However, this evaluation was not based on a random or systematic sample of the full cohort and demonstrated only moderate interobserver agreement (ICC = 0.58). Accordingly, these findings should be interpreted with caution and should not be considered a robust standalone outcome measure.

Finally, the limited availability of polyurethane-coated breast implants in certain regulatory environments, including the United States, may restrict the direct applicability of this implant-assisted approach in some geographic markets. In addition, all procedures were performed by experienced surgeons using a standardized protocol; while this enhances internal consistency, the generalizability of these results to other practice settings or varying levels of surgical expertise may be limited.

## 5. Conclusions

Within the limitations of this study, conical implants appear to be a feasible and clinically applicable option for the surgical correction of tuberous breast deformity when used as part of a standardized, anatomy-driven reconstructive approach. The observed complication and reoperation profile, together with high patient-reported satisfaction, suggests the potential clinical feasibility of this implant geometry in selected cases, particularly in mild-to-moderate deformities. As one of the first clinical series evaluating the exclusive use of conical implants in this setting, these findings should be interpreted as exploratory and hypothesis-generating, supporting further investigation of their role as a facilitating component within a comprehensive implant-assisted reconstructive framework. Further prospective, comparative, and multicenter studies with longer follow-up are required to better characterize long-term outcomes and to assess the relative performance of different implant shapes and reconstructive strategies.

## Figures and Tables

**Table 1:** Characteristics of patients and breast implants.

Variable		Value
Mean age (years)		29.8
Active smoking		2%
Age range (years)		18–58
Grolleau Classification (%)	Type I	62%
	Type II	30%
	Type III	8%
Mean Implant Volume (cc)		258.2
Implant projection (%)	Low (LO)	4%
	Moderate (MD)	74%
	High (HI)	22%

**Table 2:** Results of the BREAST-Q questionnaire. * The “Satisfaction with implants” domain has been converted from its original 2–8 scale to a 0–100 score. (↑) Higher scores indicate better outcomes; (↓) lower scores indicate better outcomes for symptom-related domains.

Domain Assessed		Average	Standard Deviation	Scale
Breast satisfaction	Survey 1	90.1	11.9	0–100 (↑ best)
	Survey 2	92.0	9.7	0–100 (↑ best)
Physical well-being of the chest	Survey 1	7.6	7.9	0–100 (↓ best)
	Survey 2	6.0	6.4	0–100 (↓ best)
Satisfaction with implants *	Survey 1	96	6.7	0–100 (↑ best)

**Table 3:** Complications following the surgical procedures. * Two of the four reoperations corresponded to elective fat grafting procedures performed to improve aesthetic contour.

Type of Complication		Cases (n = 50)	Frequency
Early postoperative complications		4	8%
Other complications in follow-up	Capsular contracture	1	2%
	Implant rupture	0	0%
Total reoperations		4 *	8%

## Data Availability

The data supporting the findings of this study are available from the corresponding author upon reasonable request.

## Supplementary Materials

The following supporting information can be downloaded at https://www.mdpi.com/article/10.3390/medicina62050930/s1: Video S1: Step-by-step demonstration of the standardized surgical technique for tuberous breast correction. Step-by-step demonstration of the standardized surgical technique for correction of tuberous breast deformity. The video illustrates preoperative markings, periareolar access, radial glandular releases, posterior myotomy, inframammary fold repositioning, correction of areolar herniation using the Puckett technique, antibiotic irrigation of the implant pocket, and placement of a conical polyurethane implant using a dual-plane II approach. The video is intended as a complementary visual aid illustrating the main operative steps of the standardized approach.

## Abbreviations

The following abbreviations are used in this manuscript:

NAC	Nipple–areola complex
MD	Moderate projection
HI	High projection
LO	Low projection
ICC	Intraclass correlation coefficient
APC	Article processing charge
