# Supplementary material for: Conical Implants in Tuberous Breast Correction: Clinical and Patient-Reported Outcomes
Source: Medicina (Kaunas). 2026 May 10;62(5):930. doi: 10.3390/medicina62050930 (PMC13208704; doi:10.3390/medicina62050930)
Supplement: Supplementary file 1 [file medicina-62-00930-s001.zip › Medicina/CEIm25-82 Informe FAVORABLE.pdf]

INFORME DEL COMITÉ DE ÉTICA DE LA INVESTIGACIÓN CON MEDICAMENTOS (CEIm) DEL HOSPITAL UNIVERSITARIO DE GETAFE

Dña. Mª Teresa Ramírez López, Vicepresidenta del Comité de Ética de la Investigación con Medicamentos del Hospital Universitario de Getafe

CERTIFICA:

Que este Comité en su reunión del día jueves, 04 de septiembre de 2025 (A08/25) ha evaluado la documentación presentada por D. Jorge González Prieto, correspondiente al Estudio observacional descriptivo y ambispectivo titulado: **"Implantes cónicos en mamas tuberosas: un estudio ambispectivo".**

- **Protocolo:** *Versión 2, 07/07/2025*
- **Hoja de Información al Participante y Consentimiento Informado:** *Versión 2, 07/07/2025*

y considera que:

- Se cumplen los requisitos necesarios de idoneidad del Protocolo en relación con los objetivos del estudio y están justificados los riesgos y molestias previsibles para el sujeto.
- La capacidad del investigador y los medios disponibles son adecuados para llevar a cabo el estudio.
- El alcance de las compensaciones económicas previstas no interfiere con el respeto de los postulados éticos.

Por ello, este Comité emite **Informe Favorable** sobre la realización de dicho Estudio observacional descriptivo y ambispectivo a, D. Jorge González Prieto, como Investigador Principal, del Servicio de Cirugía Plástica de nuestro centro.

Se recuerda al Investigador que al ejecutar este proyecto contrae una serie de compromisos con respecto al Comité recogidos en el Anexo I (hoja adjunta).

Lo que firmo en Getafe, a 04 de septiembre de 2025.

Fdo.: Dña. Mª Teresa Ramírez López
Vicepresidenta del CEIm
Hospital Universitario de Getafe

Hospital Universitario de Getafe
SaludMadrid

Dña. Mª Teresa Ramírez López, Vicepresidenta del Comité de Ética de la Investigación con Medicamentos del Hospital Universitario de Getafe.

HACE CONSTAR QUE:

Que la composición del CEIm, en la reunión en la que ha sido evaluado el Estudio observacional descriptivo y ambispectivo titulado: **"Implantes cónicos en mamas tuberosas: un estudio ambispectivo".** *Versión 2, 07/07/2025*

Es la siguiente:

Presidente	D. Óscar Peñuelas Rodríguez
Vicepresidenta	Dña. Mª Teresa Ramírez López
Secretaria Técnica	Dña. Isabel Sánchez Muñoz
Vocales	Dña. Rocío Álvarez Nido<br>Dña. Mercedes M. Cavanagh<br>Dña. Marina Carbonero García<br>Dña. Ana Isabel Castillo Varón<br>Dña. Patricia Cuenca Gómez<br>Dña. Irene Cuadrado Pérez<br>Dña. Mª Concepción García Escudero<br>Dña. Marta González Bocanegra<br>Dña. Olga Laosa Zafra<br>Dña. Teresa Molina García<br>D. Alfonso Monereo Alonso<br>Dña. Rocío Queipo Matas<br>D. Javier Sánchez-Rubio Ferrández<br>Dña. Ana Rosa Solórzano Martín

ANEXO I: Compromisos contraídos por el investigador principal con respecto al Comité de Ética de la Investigación con Medicamentos:

Se recuerda al investigador que la ejecución del estudio le supone los siguientes compromisos con el Comité:

- Ejecutar el estudio con arreglo a lo especificado en el protocolo, tanto en los aspectos científicos como en los aspectos éticos.
- Notificar al Comité todas las modificaciones o enmiendas en el estudio y solicitar una nueva evaluación de las relevantes.
- Informe de seguimiento anual (si el estudio dura menos de un año, con el informe final será suficiente).
- Enviar al Comité un informe final al término de la ejecución del estudio. Este informe deberá incluir los siguientes apartados:
    - Número de registro del estudio en bases de datos públicas de proyectos de investigación, si procede.
    - Memoria final del estudio, la enviada a las agencias reguladoras.
    - Publicación/es científica/s generada/s por el estudio.
    - Información (tipo y modo) transmitida a los sujetos del estudio sobre los resultados de la investigación y sobre los que afecten directamente a su salud (si procede).
