# Supplementary material for: Conical Implants in Tuberous Breast Correction: Clinical and Patient-Reported Outcomes
Source: Medicina (Kaunas). 2026 May 10;62(5):930. doi: 10.3390/medicina62050930 (PMC13208704; doi:10.3390/medicina62050930)
Supplement: Supplementary file 1 [file medicina-62-00930-s001.zip › Medicina/Written informed consents /DCI 4.pdf]

CONSENTIMIENTO INFORMADO PARA PUBLICACIÓN DE IMÁGENES

Título del estudio: Conical Implants in Tuberous Breast Correction: Clinical and Patient-Reported Outcomes

Yo, Celia Fernandez Fernandez, mayor de edad, con documento de identidad nº 04227696C, declaro que he sido informada de forma adecuada y comprensible sobre el uso de mis imágenes clínicas con fines científicos y académicos.

Autorizo expresamente a los autores del estudio a utilizar mis fotografías clínicas preoperatorias y postoperatorias, así como imágenes derivadas de procedimientos quirúrgicos, para su publicación en revistas científicas médicas, presentaciones académicas, material docente y otros medios relacionados con la difusión del conocimiento médico.

Entiendo que:

- Las imágenes se utilizarán únicamente con fines científicos, docentes y editoriales.
- Mi identidad no será revelada y no se incluirán datos personales identificables.
- Las imágenes podrán publicarse en formato impreso y digital, incluyendo revistas de acceso abierto.
- No recibiré ninguna compensación económica por el uso de dichas imágenes.
- Esta autorización se concede de forma voluntaria.

Declaro que comprendo plenamente el contenido de este consentimiento y que lo firmo libremente.

Fecha: 09 / 02 / 2026

Nombre completo del paciente: Celia Fernandez Fernandez

Firma del paciente: ____________________

Nombre del médico responsable: Dr. Gonzalez-Nicolas

Dr. González-Nicolás Albandea
CIRUJANO PLÁSTICO
Colegiado 31411 Madrid
Miembro Numerario SECPRE 835

Firma del médico: ____________________
