# Supplementary material for: Conical Implants in Tuberous Breast Correction: Clinical and Patient-Reported Outcomes
Source: Medicina (Kaunas). 2026 May 10;62(5):930. doi: 10.3390/medicina62050930 (PMC13208704; doi:10.3390/medicina62050930)
Supplement: Supplementary file 1 [file medicina-62-00930-s001.zip › Medicina/Written informed consents /DCI 5.pdf]

INFORMED CONSENT FOR IMAGE PUBLICATION

Study Title: Conical Implants in Tuberous Breast Correction: Clinical and Patient-Reported Outcomes

I, Regina Rios, of legal age, holder of identification document number 874751977-00 hereby declare that I have been adequately and clearly informed about the use of my clinical images for scientific and academic purposes.

I expressly authorize the authors of the study to use my preoperative and postoperative clinical photographs, as well as images derived from surgical procedures, for publication in medical scientific journals, academic presentations, educational materials, and other media related to the dissemination of medical knowledge.

I understand that:

- The images will be used exclusively for scientific, educational, and editorial purposes.
- My identity will not be disclosed, and no personally identifiable data will be included.
- The images may be published in print and digital formats, including open-access journals.
- I will not receive any financial compensation for the use of these images.
- This authorization is granted voluntarily.

I declare that I fully understand the content of this consent and that I sign it freely.

Date: October / 2nd / 2025

Patient's full name: Regina Lúcia Rios

Patient's signature:

Name of responsible physician: Bárbara Helena Barcaro Machado

Physician's signature:
