# Supplementary material for: Conical Implants in Tuberous Breast Correction: Clinical and Patient-Reported Outcomes
Source: Medicina (Kaunas). 2026 May 10;62(5):930. doi: 10.3390/medicina62050930 (PMC13208704; doi:10.3390/medicina62050930)
Supplement: Supplementary file 1 [file medicina-62-00930-s001.zip › Medicina/Written informed consents /Informed_consent_publication_images_EN.pdf]

INFORMED CONSENT FOR PUBLICATION OF CLINICAL IMAGES

Title of the study: Conical Implants in Tuberous Breast Correction: Clinical and Patient-Reported Outcomes

I, ____, confirm that I have been adequately informed about the use of my clinical images for scientific and academic purposes.

I expressly authorize the authors of the above-mentioned study to use my preoperative and postoperative clinical photographs, as well as images derived from surgical procedures, for publication in scientific medical journals, academic presentations, educational materials, and other formats related to the dissemination of medical knowledge.

I understand that:

- My images will be used exclusively for scientific, educational, and editorial purposes.
- My identity will not be disclosed and no personally identifiable information will be published.
- The images may be published in printed and digital formats, including open-access journals.
- I will receive no financial compensation for the use of these images.
- This authorization is given voluntarily.

I confirm that I fully understand the content of this consent and that I sign it freely.

Date: ____ / ____ / ______

Patient's full name: ________________________________

Patient's signature: ________________________________

Name of responsible physician: ________________________________

Physician's signature: ________________________________
